# Effect of pH on solubility of white Mineral Trioxide Aggregate and Biodentine: An in vitro study

**DOI:** 10.15171/joddd.2018.031

**Published:** 2018-09-18

**Authors:** S Pushpa, Chakit Maheshwari, Garima Maheshwari, N Sridevi, Puneeta Duggal, Puneet Ahuja

**Affiliations:** Department of Conservative Dentistry and Endodontics, Rama Dental College-Hospital and Research Centre, Kanpur - 208024, Uttar Pradesh, India

**Keywords:** Biodentine, calcium silicate cements, endodontic inflammation, mineral trioxide aggregate, solubility

## Abstract

***Background.*** The aim of this study was to evaluate the effect of acidic, neutral and alkaline environments on the solubility of white mineral trioxide aggregate (WMTA) and Biodentine (BD).

***Methods.*** Thirty-nine ring molds were randomly divided into three groups of A, B, and C (n = 12) with pH values of 7.4, 4.4 and 10.4, respectively, and an empty mold was used as a control. Each group was further divided into two subgroups (1 and 2) according to the material studied. The samples in groups A, B and C were transferred into synthetic tissue fluid buffered at pH values of 7.4, 4.4 and 10.4, respectively, and kept in an incubator at 37°C with 100% humidity. Daily solubility at 1-, 2-, 5-, 14-, 21-, and 30-day intervals and cumulative solubility up to 5-, 14-, and 30-day intervals were calculated. Statistical analysis was carried out with independent-samples t-test, two-way ANOVA and post hoc Tukey tests using SPSS 18. Statistical significance was set at P<0.05.

***Results.*** Both WMTA and BD exhibited the highest solubility in acidic pH with 5.4235±0.1834 and 10.7516±0.0639 mean cumulative solubility values at 30-day interval, respectively. At all exposure times, BD was significantly more soluble than WMTA (P<0.001).

***Conclusion.*** Acidic periapical environment jeopardized the solubility of both WMTA and BD, affecting their sealing characteristics in clinical applications like perforation repair procedures and blunderbuss canals.

## Introduction

It has been well established that leakage of irritants into the periapical tissues accounts for most of the endodontic failures.^1^ An ideal endodontic repair material therefore must seal the pathways of communication between the root canal system and its surrounding tissues.A plethora of dental restorative materials like amalgam, composites, gold-foil, zinc oxide eugenol-based cements and calcium silicate-based materials have been extensively investigated for use as endodontic repair materials.^2^

Attempt to develop a material with “ideal” characteristics led to the introduction of mineral trioxide aggregate (MTA) by Torabinejad et al in 1993.^3^ Its main components are tricalcium silicate, tricalcium aluminate, and oxides of silicates and bismuth. MTA has many favorable properties that support its clinical applications like pulp capping, pulpotomy, apexogenesis, apexification, repair of root perforations, and as a root-end repair material.^2,4^ Despite the good physicochemical and biological properties, MTA has some shortcomings such as difficult handling characteristics, tooth discoloration, lower compressive and flexural strengths than dentin and high cost.^5^ The long setting time also favors its solubility and disintegration from root-end cavities.^6,7^

In 2010, a new calcium silicate-based material, Biodentine (BD), was introduced by Gilles and Olivier. BD is composed of tricalcium silicate, calcium carbonate, zirconium oxide and a liquid containing calcium chloride (CaCl_2_) as setting accelerator. It is a fast-setting material which is claimed to be suitable for use as a dentin substitute and for endodontic applications comparable with MTA.^8^

In various clinical applications such as root-end filling and repair of root perforations, MTA and BD are frequently applied in contact with tissue fluids such as serum and blood. The adjacent tissue fluid might have normal or lower pH levels because of infection and inflammation.^9^ On the contrary, if the inflammation in the periapical tissue is decreased by endodontic treatment, the pH will become slightly alkaline (pH = 7.4) within 7 days or less.^6^ Therefore, during the setting process the materials surface is exposed to acidic or slightly alkaline pH levels.Studies have reported that push-out bond strength of MTA could be influenced by different alkaline pH values. Also placement in an acidic environment increases the solubility of WMTA.^10^ Some authors have reported that BD exhibited increased solubility, prolonged alkalinity and increased calcium release than MTA when stored in deionized water at different immersion periods.^8,11^

Lack of solubility is a desired characteristic for endodontic repair cements. ISO 6876:2001 standard places the acceptable limit of weight loss for solubility test at 3%.^12^ Higher solubility of cements will result in leaching of components from endodontic space that might exert undesirable biologic effects on surrounding tissues.^13^ The effect of pH on the solubility of MTA has been studied.^10^ However, sufficient literature on the effect of pH on solubility of BD is lacking; therefore, the aim of this study was to compare the solubility of WMTA and BD in synthetic tissue fluid (STF) at pH values of 4.4, 7.4 and 10.4. The null hypothesis stated that there is no difference in the solubility of both the tested materials under any environmental pH.

## Methods

**Figure 1:**
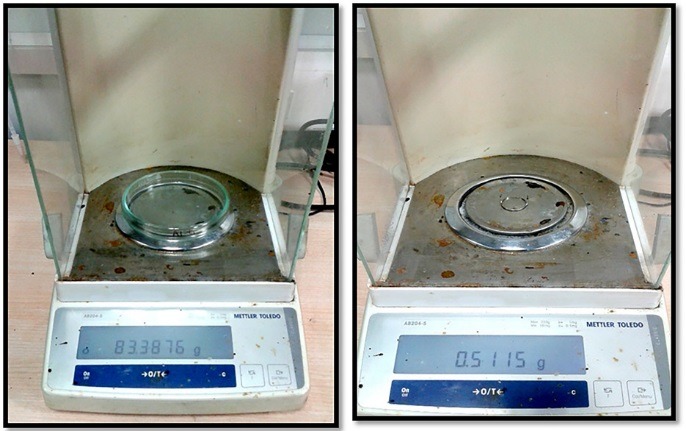
Analytical balance (Mettler Toledo) used for measuring Petri dishes and ring molds.

The solubility of WMTA (PROROOT^TM^, Dentsply International Inc., York, PA, USA) and BD (Biodentine^TM^, Septodont, Saint Maur des Fosses, France) were determined according to the method recommended by the ISO specification 6876:2001 and ADA specification #30.^12^ Thirty-nine stainless steel ring molds (internal diameter: 20±0.1 mm; height: 1.5±0.1 mm) were cleaned with acetone (SD Fine Chem, Maharashtra, India) in an ultrasound bath (Clean 120-HD) for 15 minutes and air-dried for 30 minutes. Two stainless-steel wires were fixed at the mold in order to hang the samples in a glass Petri dish (Borosil; diameter: 90 mm, volume: 100 mL) so that the surfaces did not contact and the materials were not disrupted in the dish. All the molds were weighed in an analytical balance (accuracy: 0.0001 g) (Mettler Toledo, Ohio, United States) three times before use to record the average reading and recorded as dry ring weight (DRW; Figure 1). The same analytical balance was used throughout the experiment.

**Figure 2:**
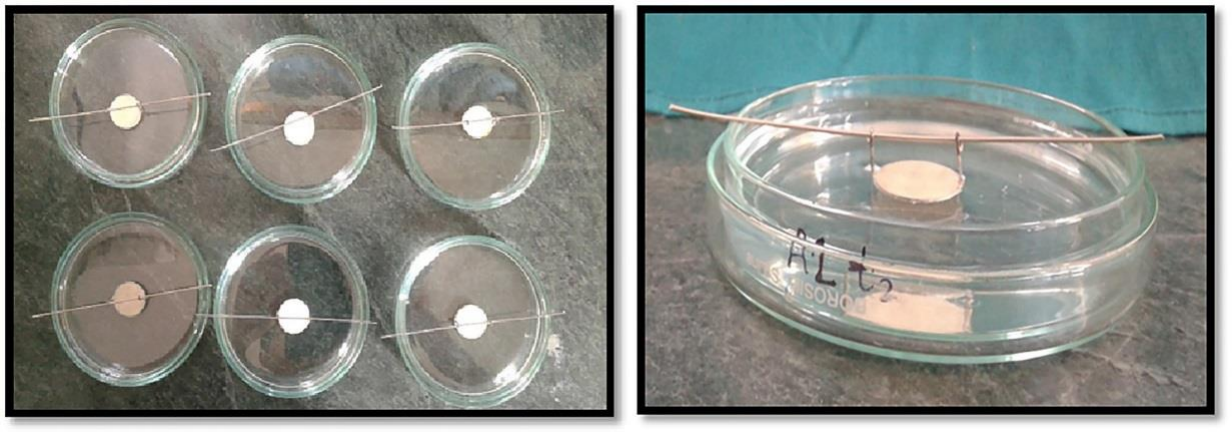
The ample rings prepared with WMTA and BD.

The samples were divided into three groups A, B, and C (n=12) and an empty mold was used as a control. Every experimental group was further subdivided into two subgroups: 1 and 2. WMTA was used in the first subgroups of each group (A1, B1 and C1), while in the second subgroups (A2, B2 and C2) BD was used (Figure 2). Each material was mixed and placed according to manufacturers' instructions. A single operator performed all the manipulations and care was taken to avoid incorporation of air voids or leakage of the materials from the molds.

The samples in groups A, B and C were soaked in STF buffered with potassium hydroxide at pH=7.4, with butyric acid at pH=4.4, and with potassium hydroxide at pH=10.4, respectively, using a 4×2-cm piece of gauze. They were then incubated at 37°C and 100% relative humidity for 24 hours. Subsequently, the samples were air-dried for 15 minutes. Each sample was weighed three times to record the average reading and was noted as initial dry weight (IDW). Sample weight (SW) was calculated by subtracting the mean DRW from the mean IDW.

### Preparation of the acidic, neutral and alkaline synthetic tissue fluids

STF was prepared by dissolving the following solutes in 10 liters of water: 1.7 g of monopotassium phosphate, 11.8 g of disodium phosphate, 2 g of potassium chloride and 80 g of sodium chloride. The pH of STF in groups A and C was adjusted at 7.4 and 10.4, respectively, with potassium hydroxide, and in group B at 4.4 with butyric acid using a digital pH meter (Keroy Pvt. Ltd., Uttar Pradesh, India).

### Measurement of solubility

**Figure 3:**
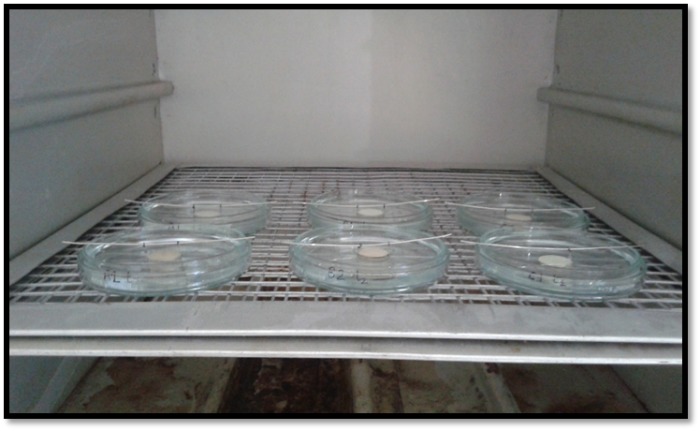
The samples placed in desiccator.

**Figure 4:**
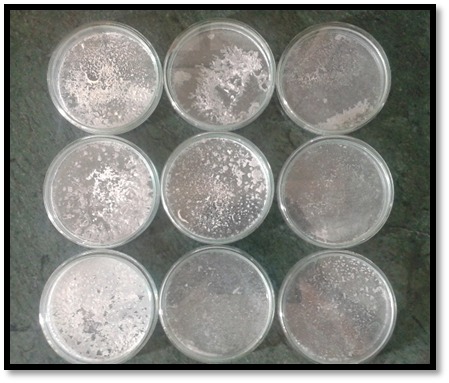
Dried samples containing residue

Individual weights of 39 dried and labelled Petri dishes were recorded as dry dish weights. In group A, each sample was transferred into a Petri dish containing 50 mL of STF at pH=7.4. The dishes were incubated at 37°C and 100% humidity and were retrieved from the incubator (Mahendra Scientific, Uttar Pradesh, India) at 1-, 2-, 5-, 14-, 21-, and 30-day intervals. After the specific immersion period, the samples were hanged over Petri dishes and gently rinsed with 15 mL of de-ionized water (Lab Chem, Uttar Pradesh, India) to collect the residues. The rinse water in the dishes was evaporated at a temperature slightly below the boiling point (70°C). The dishes were then dried in an oven at 105°C and cooled down in the same desiccator (Mahendra Scientific, Uttar Pradesh, India) (Figure 3). Each petri dish was individually weighed to record dry residue weight (Figure 4). The amount of components isolated from the samples, i.e. the residue weight, was calculated using Equation 1:

RW = DRsW − (mean DDW + Solute weight)

where RW is residual weight, DRsW is dry residue weight and DDW is dry dish weight.

Figure 4. Dried samples containing residue

In group B, each sample was transferred into a dish containing 50 mL of STF at pH=4.4 and in group C at pH=10.4. The dry material was measured at 1-, 2-, and 5-day intervals. From the 5th day, the samples were placed in a dish containing STF at pH=7.4. The RW was measured in a manner similar to that in group A. Solubility values pertaining to different periods of time were recorded in two different ways as daily solubility and cumulative solubility.

Daily solubility of each sample was calculated by Equation 2:

Daily solubility = RW/SW × 100

The sum of all the values of all the time intervals was reported as the cumulative solubility, with the sum at 30-day interval being reported as the total solubility of the material. At the end of the 30th day all the samples were again placed in a vacuum desiccator for 4 hours, followed by a 21-hour period in an oven at 105°C. The samples were then weighed and weight changes of all the samples were calculated in comparison to baseline weights.

As controls, 3 empty sample molds together with soldered stainless steel wires were immersed in STF at pH values of 4.4, 7.4 and 10.4 for 30 days and any change in weight was noted. Statistical analysis was performed using SPSS 18. A P-value of <0.05 was considered as statistically significant. Comparison of the mean daily solubility between the two subgroups (A1 & A2; B1 & B2; C1 & C2) was carried out with independent-samples t-test. Two-way ANOVA with post hoc Tukey tests was applied to compare the mean cumulative solubility of WMTA and BD in three respective pH values i.e., 4.4, 7.4 and 10.4.

## Results

BD exhibited significantly higher solubility than WMTA (P<0.001) in all the environments, i.e. neutral (pH=7.4), acidic (pH=4.4) and alkaline (pH=10.4) (Tables 1) to 3)). However, there was a statistically insignificant difference at 14-day interval in the alkaline environment (P=0.182). The negative values represent an increase in weight.

**Table 1:** Comparison of the means and standard deviations of the daily solubility for group A (pH=7.4) at different time intervals expressed in percentages

	**t**_1_		**t**_2_		**t**_5_		**t**_14_		**t**_21_		**t**_30_	
	**Mean**	**SD**	**Mean**	**SD**	**Mean**	**SD**	**Mean**	**SD**	**Mean**	**SD**	**Mean**	**SD**
**A1 (WMTA)**	.5023	.0436	1.1861	.0466	-2.1761	.0489	-3.7543	.0420	-3.9887	.0561	-4.2231	.0511
**A2 (BD)**	.7213	.0362	1.9358	.0303	2.6981	.0265	-.6244	.0133	-1.5288	.0242	-2.2997	.0233
**p-value**	<0.001; Sig		<0.001; Sig		<0.001; Sig		<0.001; Sig		<0.001; Sig		<0.001; Sig	

BD, Biodentine; SD, standard deviation; t, time interval; WMTA, white mineral trioxide aggregate

**Table 2:** Comparison of the means and standard deviations of the daily solubility for group B (pH=4.4) at different time intervals expressed in percentages

	**t**_1_		**t**_2_		**t**_5_		**t**_14_		**t**_21_		**t**_30_	
	**Mean**	**SD**	**Mean**	**SD**	**Mean**	**SD**	**Mean**	**SD**	**Mean**	**SD**	**Mean**	**SD**
**B1 (WMTA)**	2.9400	.0713	3.1558	.0661	4.7913	.0640	-.9984	.0446	-2.0670	.0728	-2.3982	.0529
**B2 (BD)**	3.2322	.0498	3.8117	.0331	5.1231	.0321	1.5529	.0367	-1.0514	.0359	-1.9170	.0580
**p-value**	<0.001; Sig		<0.001; Sig		<0.001; Sig		<0.001; Sig		<0.001; Sig		<0.001; Sig	

BD, Biodentine; SD, standard deviation; t, time interval; WMTA, white mineral trioxide aggregate

**Table 3:** Comparison of the means and standard deviations of the daily solubility for group C (pH=10.4) at different time intervals expressed in percentages

	**t**_1_		**t**_2_		**t**_5_		**t**_14_		**t**_21_		**t**_30_	
	**Mean**	**SD**	**Mean**	**SD**	**Mean**	**SD**	**Mean**	**SD**	**Mean**	**SD**	**Mean**	**SD**
**C1 (WMTA)**	.5036	.0537	.8687	.0490	-.2772	.0526	-1.1967	.0407	-2.2825	.0532	-2.8556	.0531
**C2 (BD)**	.6498	.0570	1.8088	.0427	2.0028	.0392	-1.1590	.0500	-1.9737	.0424	-2.5653	.0572
**p-value**	0.001; Sig		<0.001; Sig		<0.001; Sig		0.182; NS		<0.001; Sig		<0.001; Sig	

BD, Biodentine; NS, non significant; SD, standard deviation; t, time interval; WMTA, white mineral trioxide aggregate

**Figure 5:**
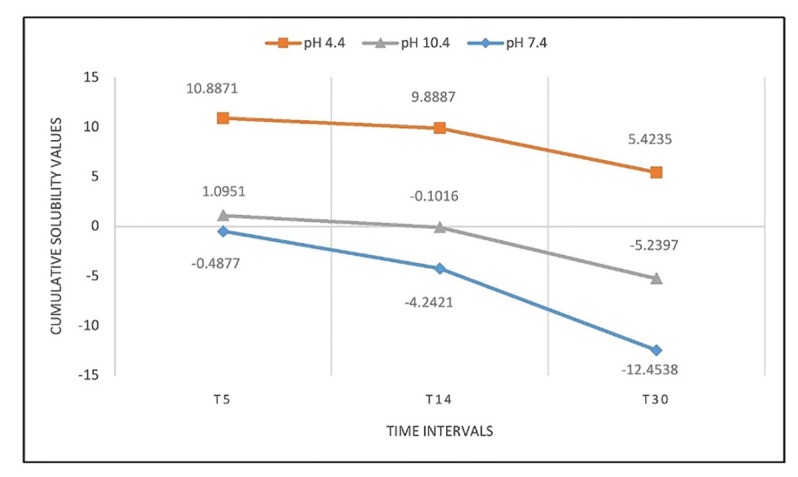
. Cumulative solubility values of WMTA up to 5 days, 14 days and 30 days interval at pH 4.4, 7.4, 10.4.

**Figure 6:**
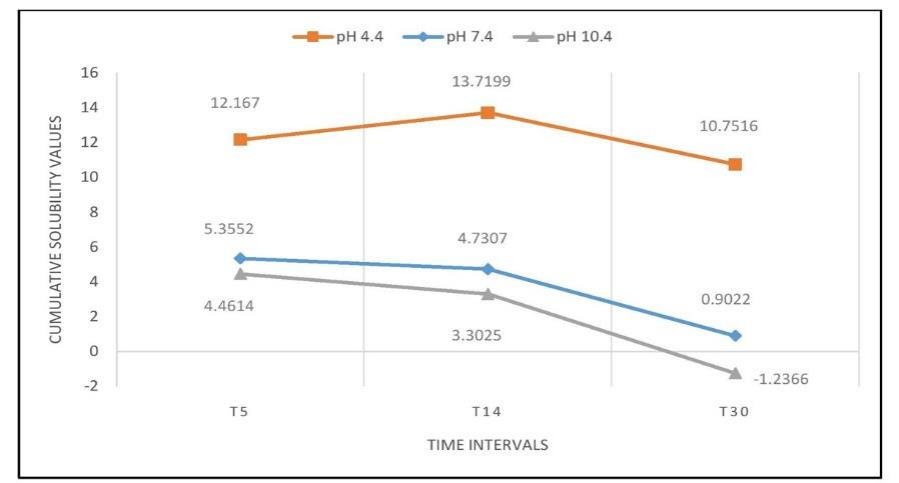
Cumulative solubility values of BD up to 5 days, 14 days and 30 days interval at pH 4.4, 7.4, 10.4.

Cumulative solubility was also calculated at 5-, 14- and 30-day intervals. WMTA exhibited more solubility in the acidic environment followed by the alkaline environment, with the least the in neutral environment (P<0.001; Figure 5). However, BD exhibited more solubility in the acidic environment followed by the neutral environment, with the least in the alkaline environment (P<0.001; Figure 6).

After 30 days, an increase in mass was detected for WMTA in both alkaline and neutral environments (-12.4538±0.0742 and -5.2397 ± 0.1678, respectively). On the contrary, BD exhibited an increase in mass only in the alkaline environment (-1.2366±0.0898) after 30 days.

## Discussion

Sealing potential and dimensional changes of an endodontic repair material is directly related to its solubility, which leaves spaces that might favor bacterial colonization and their passage into periapical tissues.^14^ Our results demonstrated that BD was significantly more soluble than WMTA in all the environmental pH conditions.It might be speculated that BD releases a higher amount of calcium ions in the STF buffer, which might explain its higher solubility compared to WMTA. However, this is in contrast with a study by Bortoluzzi et al,^15^ in which CaCl_2_ reduced the setting time and solubility of WMTA without promoting its disintegration. Considering the similarities between ProRoot MTA and BD, one would expect similar results from BD.

In our study, the samples were placed in STF with pH values of 7.4, 4.4 and 10.4 during initial setting in order to simulate physiologic and pathologic conditions to evaluate WMTA and BD solubility. Butyric acid was used to produce an acidic pH in group B, which is a by-product of anaerobic bacteria metabolism.^16^ Since subsequent to therapeutic interventions and elimination of the inflamed tissue, the tissue pH returns to that of a neutral environment, the samples in groups B and C were placed in the acidic and alkaline environments, respectively, for only 5 days.

The results of our study also coincides with studies done by Saghiri et al^17^ and Yavari et al,^10^ in which all the tested materials showed the highest solubility in the acidic environment. To explain this we must note that both WMTA and BD undergoe structural changes in low pH values, resulting in altered sealing ability.This acidic environment can cause acid corrosion in which calcium hydroxide (Ca(OH)_2_) and the calcium sulfoaluminate phases decompose and produce porosities.^17^ Since porosity results in the progression of solubility, increased WMTA and BD solubility in the present study might be attributed to increased porosity and changes in WMTA and BD crystalline structure after exposure to an acidic environment.

Previous studies have evaluated the solubility of MTA and BD in distilled water, which was approximately 3% less than that determined by ISO specification.^11,18^ STF was used in this study instead of distilled water for a better evaluation of bioactive components which dissociate from calcium silicate cements at a higher rate in STF.^10,19^ Calcium ions released from calcium silicate cements react with the phosphate in the STF buffer to form hydroxyapatite. The negative readings in the present study might be due to formation of hydroxyapatite and hydration of WMTA and BD and indicate an increase in weight. Nearly all the WMTA samples absorbed mass from STF buffer after 5 days in all the environmental pH conditions except in acidic pH. In the BD group this was only observed after 14 days. However, these results are not consistent with the study by Kaup et al,^11^ who observed an increase in weight of WMTA at all the experimental time intervals and for BD after 28 days. This variation might be attributed to the compositional difference of STF used in our study compared to that of phosphate-buffered saline (PBS) used in their study.

The long setting time of MTA favors its solubility and/or displacement from the retrograde cavity.^20^ When set MTA contacts tissue fluids, calcium and hydroxyl ions are released from Ca(OH)_2_ molecule, raising the pH to approximately 12.5.^11^ These alkaline pH levels and calcium ions detected in the periapical tissues surrounding MTA are also considered essential to hard tissue deposition.^21^ Researchers have found that BD also releases significantly higher amounts of calcium ions compared to MTA when immersed in PBS.^22^ The high amount of calcium ion release from BD can be correlated to the presence of a calcium silicate component and calcium chloride in the material.^23^

Although the procedure for ascertaining solubility closely resembles the clinical situation, the results can only be partly extended to in vivo situation. However, only a small amount of cement comes in contact with periapical fluids contrary to our study, in which the surface area was exposed to the periapical environment at a higher rate. All the materials were examined for solubility after they fully set; therefore, these test conditions were different from clinical situation where the materials are used before their initial setting.

In a study by Steinig et al,^24^ one-visit apexification protocol with MTA as an option to the traditional multiple calcium hydroxide treatments was proposed. However, according to our study acidic environment increased the solubility of WMTA and BD well above the ISO 6876:2001 standards,^12^ and in such cases a multiple-visit treatment with some intracanal medicament is advisable to neutralize the periapical pH and also to prevent early dissolution of WMTA or BD.A greater width of WMTA and BD is also recommended in cases such as periapical surgery, perforation repair and external root resorption repair because the tissue humidity promotes partial dissolution of the material, which extends up to 14 days according to our results.^10^

## Conclusion

Within the limits of this in vitro study, it can be concluded that both WMTA and BD fulfilled the requirements of ISO 6876:2001, i.e. solubility <3% after 24 hours in all the environmental pH conditions, except BD which exhibited solubility >3% in the acidic pH.

## Acknowledgments

Not applicable.

## Author Contributions

The study was planned by SP and CM. CM carried out the laboratory procedures. The statistical analyses and interpretation of data were carried out by PD, PA and NS. CM and GM were responsible for literature search and manuscript preparation. SP, PD and NS critically revised the manuscript for intellectual content. All the authors contributed to the final draft and have read and approved the final manuscript.

## Funding

Not applicable.

## Competing interests

The authors declare that they have no competing interests with regards to authorship and/or publication of this paper.

## Ethics approval

Not applicable.
